# Amisulpride augmentation therapy improves cognitive performance and psychopathology in clozapine-resistant treatment-refractory schizophrenia: a 12-week randomized, double-blind, placebo-controlled trial

**DOI:** 10.1186/s40779-022-00420-0

**Published:** 2022-10-18

**Authors:** Ming-Huan Zhu, Zhen-Jing Liu, Qiong-Yue Hu, Jia-Yu Yang, Ying Jin, Na Zhu, Ying Huang, Dian-Hong Shi, Min-Jia Liu, Hong-Yang Tan, Lei Zhao, Qin-Yu Lv, Zheng-Hui Yi, Feng-Chun Wu, Ze-Zhi Li

**Affiliations:** 1 Clinical Research Center for Mental Disorders, School of Medicine, Shanghai Pudong New Area Mental Health Center, Tongji University, Shanghai, 200124 China; 2 Qingdao Mental Health Center, Qingdao, 266034 Shandong China; 3 Shanghai Mental Health Center, Shanghai Jiao Tong University School of Medicine, Shanghai, 200030 China; 4 Department of Psychiatry, the Affiliated Brain Hospital of Guangzhou Medical University, Guangzhou, 510370 China; 5 Guangdong Engineering Technology Research Center for Translational Medicine of Mental Disorders, Guangzhou, 510370 China

**Keywords:** Schizophrenia, Clozapine-resistant treatment refractory schizophrenia, Clozapine, Amisulpride, Augmentation

## Abstract

**Background:**

Although clozapine is an effective option for treatment-resistant schizophrenia (TRS), there are still 1/3 to 1/2 of TRS patients who do not respond to clozapine. The main purpose of this randomized, double-blind, placebo-controlled trial was to explore the amisulpride augmentation efficacy on the psychopathological symptoms and cognitive function of clozapine-resistant treatment-refractory schizophrenia (CTRS) patients.

**Methods:**

A total of 80 patients were recruited and randomly assigned to receive initial clozapine plus amisulpride (amisulpride group) or clozapine plus placebo (placebo group). Positive and Negative Syndrome Scale (PANSS), Scale for the Assessment of Negative Symptoms (SANS), Clinical Global Impression (CGI) scale scores, Repeatable Battery for the Assessment of Neuropsychological Status (RBANS), Treatment Emergent Symptom Scale (TESS), laboratory measurements, and electrocardiograms (ECG) were performed at baseline, at week 6, and week 12.

**Results:**

Compared with the placebo group, amisulpride group had a lower PANSS total score, positive subscore, and general psychopathology subscore at week 6 and week 12 (*P*_Bonferroni_ < 0.01). Furthermore, compared with the placebo group, the amisulpride group showed an improved RBANS language score at week 12 (*P*_Bonferroni_ < 0.001). Amisulpride group had a higher treatment response rate (*P* = 0.04), lower scores of CGI severity and CGI efficacy at week 6 and week 12 than placebo group (*P*_Bonferroni_ < 0.05). There were no differences between the groups in body mass index (BMI), corrected QT (QTc) intervals, and laboratory measurements. This study demonstrates that amisulpride augmentation therapy can safely improve the psychiatric symptoms and cognitive performance of CTRS patients.

**Conclusion:**

This study indicates that amisulpride augmentation therapy has important clinical significance for treating CTRS to improve clinical symptoms and cognitive function with tolerability and safety.

*Trial registration* Clinicaltrials.gov identifier- NCT03652974. Registered August 31, 2018, https://clinicaltrials.gov/ct2/show/NCT03652974

## Background

Schizophrenia is a severe psychiatric disorder characterized by positive symptoms, negative symptoms, and cognitive deficits [1, 2]. Despite a wide variety of available antipsychotic drugs, there are still many schizophrenia patients (about 1/5 to 1/3) who are resistant to two or more antipsychotic treatments, defined as “treatment-resistant schizophrenia (TRS)” or “treatment-refractory schizophrenia” [3, 4].

Clozapine is the only evidence-based antipsychotic drug for treating TRS patients [5, 6]. However, even with sufficient clozapine levels in the blood, about 1/3 to 1/2 of TRS patients are still resistant to clozapine [7, 8]. According to the definition of TRS proposed by Kane [9] and Honer et al. [10], TRS patients who do not respond well to clozapine monotherapy are known as having clozapine-resistant treatment-refractory schizophrenia (CTRS). According to the National Institute for Health and Care Excellence guidelines for treating TRS [11], augmentation therapies may have potential benefits for TRS patients who do not respond to clozapine monotherapy [12]. Previous studies have shown that when dopamine D2 receptors are 70% or more occupied, antipsychotics achieve their maximum efficacy [13, 14]. Clozapine is an antipsychotic drug with multi-receptor blocking effects, and its affinity for dopamine D2 receptors is low [15, 16]. Amisulpride has highly selective blocking effects on dopamine D2 and dopamine D3 receptors [17]. The unique dopamine receptor blocking effects of amisulpride can selectively enhance the limited dopamine D2 receptor blocking effects of clozapine [18, 19], making it a suitable drug for combination with clozapine [19]. In addition, a previous meta-analysis has shown that the efficacy of amisulpride is second only to clozapine and that its treatment interruption rate is the lowest among the 15 antipsychotics commonly used for schizophrenia treatment [20]. Previous randomized, double-blind, placebo-controlled trials using amisulpride augmented with clozapine in TRS patients showed no statistical advantages [19, 21]. However, these studies had relatively small sample sizes, and their patients may not have met CTRS criteria for the following reasons: 1) patients participating in the study may not have actually received two antipsychotic agents with different mechanisms of action in the past five years, or patients may not have taken the appropriate dose for a sufficient period of time before clozapine treatment; and 2) the studies stipulated that clozapine monotherapy should be administered for at least 3 months instead of 6 months. Previous studies have shown that some patients may have a delayed response to clozapine. Among these patients, 30% respond after 6 weeks, 20% respond after 3 months, and 10–20% respond after 6 months [22, 23], suggesting that clozapine resistance should ideally be measured after 6 months. The evidence for amisulpride combined with clozapine in the treatment of CTRS patients remains poor. Most importantly, to the best of our knowledge, few studies have investigated the effects of amisulpride augmentation on cognitive function of CTRS patients. It has been reported that 98% of patients with schizophrenia have cognitive impairment, including first-onset or chronic episode patients [24, 25]. The recovery of cognitive function is considered to be one of the main goals of clinical treatment of schizophrenia [26, 27].

Thus, this 12-week, randomized, double-blind, placebo-controlled study aims to investigate the efficacy and safety of amisulpride augmentation therapy in CTRS patients who have received at least two appropriate doses of antipsychotics with different chemical structures within a sufficient period of time and have recently received a stable dose of clozapine (i.e., at least 400 mg or more per day) for at least 6 months. The main purpose of this study was to investigate whether amisulpride augmentation therapy improved the psychopathological symptoms and cognitive performance of these CTRS patients.

## Methods

The study is a randomized, double-blind, placebo-controlled trial. Regulatory approvals for this study were obtained from the Institutional Review Board of Shanghai Pudong New Area Mental Health Center (No. 2018008), and each written informed consent was signed. The protocol was registered before participant enrolment on clincialtrials.gov (ID: NCT03652974).

### Participants

All participants were recruited from the Shanghai Pudong New Area Mental Health Center between September 6, 2018, and August 1, 2021. The inclusion criteria were: (1) Han Chinese ethnicity; (2) between 18 and 65 years old; (3) satisfied the diagnostic criteria for schizophrenia according to the Diagnostic and Statistical Manual of Mental Disorders, Fourth Edition (DSM-IV), using the Structured Clinical Interview for DSM-IV (SCID-I/P); (4) had received at least two antipsychotic agents with different mechanisms of action, at appropriate doses for a sufficient course of treatment, and had recently received a stable dose of clozapine (i.e., at least 400 mg/d or more for at least 6 months) in order to ensure a reasonable response to clozapine monotherapy; (5) a review of the patient’s past medical history revealed that the patient had stubborn psychotic symptoms and had never been effectively controlled; and (6) the patient had a baseline PANSS score > 60 before entering the study [28]. The exclusion criteria were: (1) any other major Axis I disorder; (2) serious physical diseases; (3) substance abuse/dependence; or (4) pregnant women.

### Intervention procedures

After the enrollment was completed, all eligible CTRS patients continued to take clozapine (doses from 400 to 550 mg) and were randomly assigned to receive clozapine plus amisulpride or clozapine plus placebo on a 1:1 basis. Randomization was carried out according to computer-generated random identification. The titration started with amisulpride 200 mg/d or 1 placebo tablet in the first week, amisulpride 400 mg/d or 2 placebo tablets in the second week, and up to 800 mg of amisulpride or 4 placebo tablets for the remaining 10 weeks.

Patients suffering from severe anxiety or insomnia were treated with benzodiazepines over a short time. Diphenylethyl hydrochloride was applied for a limited time in patients with extrapyramidal symptoms (EPS). No other antipsychotics and antidepressants were allowed during this study. The amisulpride and placebo tablets were identical in appearance. All researchers and participants were masked for treatment randomization and assessments.

The assessments of Positive and Negative Syndrome Scale (PANSS), Scale for the Assessment of Negative Symptoms (SANS), Repeatable Battery for the Assessment of Neuropsychological Status (RBANS), Clinical Global Impression (CGI), and Treatment Emergent Symptom Scale (TESS) were evaluated at baseline, week 6, and week 12. The primary outcome was the PANSS scores at week 6 and week 12. The secondary outcome was the responder rate, SANS, RBANS, and CGI scores at week 6 and week 12. The treatment response was determined by a more than 25% reduction in the PANSS total score [29, 30]. The PANSS reduction rate was calculated using the following formula: (baseline PANSS total score − follow-up PANSS total score)/(baseline PANSS total score − 30) × 100% [31].

### Clinical assessments

The PANSS was applied to assess psychiatric symptoms. Negative symptoms were evaluated using the SANS. The RBANS was applied to assess cognitive performance. The Clinical Global Impression severity (CGI-S), CGI improvement (CGI-I), and CGI efficacy (CGI-E) were applied to assess the symptom severity, treatment responses, and treatment effects. The TESS was applied to evaluate adverse events related to treatment. All psychiatrists were trained in the administration of the assessments, and inter-rater correlation coefficients were all above 0.8.

### Laboratory measurement, physical examination, and electrocardiogram (ECG), during the clinical trial

Laboratory measurements, physical examinations, and ECG were performed at baseline, the 6th week timepoint, and the 12th week timepoint. After an overnight fast, blood samples were collected to detect serum clozapine levels, carry out routine blood analysis, obtain a lipid profile, and measure glucose, liver, and renal function. Serum clozapine level was assayed by high performance liquid chromatography. Routine blood analysis was estimated by white blood cell (WBC), neutrophils, red blood cell (RBC), hemoglobin (Hb) and platelet (PLT), measured using a Sysmex hematology analyzer with its supporting reagents. Lipid profiles were estimated by triglyceride (TG), total cholesterol (TC), high density lipoprotein cholesterol (HDLC) and low density lipoprotein cholesterol (LDLC). TG, TC and HDLC were measured using enzymatic assay Kit (Zybio). HDLC was measured using low density lipoprotein assay Kit (Gcell). Glucose was measured using enzymatic assay Kit (Zybio). Liver function was estimated by alaninetransaminase (ALT) and aspartate transaminase (AST), measured using enzymatic assay Kit (Zybio). Renal function was estimated by serum creatinine, blood urea nitrogen (BUN) and uric acid, measured using enzymatic assay Kit (Zybio). All laboratory measurements were carried out according to the protocol provided by the manufacturer. ECG was performed using EDAN ECG machine (SE-1010).

### Statistical analysis

The distribution of the data was detected through the Kolmogorov–Smirnov one-sample test. The balance of baseline demographic and clinical characteristics between groups was compared using the chi-square test and analysis of variance (ANOVA). The qualitative data were presented as percentages and the quantitative data were expressed as mean ± standard deviation (SD). An intent-to-treat (ITT) analysis was used for sensitivity purposes, and the principle of last-observation-carrying-forward (LOCF) was used to deal with missing data.

In the beginning, repeated-measure multivariate analysis of variance (RM MANOVA) was applied to obtain the overall *P* value of PANSS and RBANS scores, respectively. Then, a repeated-measures analysis of variance (RM ANOVA) was used to examine each score of PANSS and RBANS, respectively, setting between-group factors (amisulpride and placebo) and within-group factors (baseline, week 6, and week 12), while also adjusting for confounding covariates. An RM ANOVA was conducted to measure every item of the PANSS subscale in the amisulpride group. An RM ANOVA was conducted to measure SANS scores, CGI scores, TESS total scores, body mass index (BMI), corrected QT (QTc) interval, and each laboratory measurement index, respectively. After performing an RM ANOVA, a follow-up significant multivariate omnibus test was performed, and each univariate effect was detected using an analysis of covariance (ANCOVA). If the group × time interaction was not significant, no statistical testing was further needed. If the group × time interaction had significance, an ANCOVA was used to analyze the group differences at week 6 and week 12, setting baseline score, BMI, age, sex, disease course, and baseline clozapine serum level as covariates. Bonferroni corrections were applied to correct for multiple tests. *P* values < 0.05 were considered statistically significant. Based on the power and sample size calculation at the 2-tailed 5% significance level, a sample size of 34 per group (total *n* = 68) yielded 80% of the power to detect significant differences in the primary outcome. In this study, we assumed that the dropout rate was less than 15%. PASW Statistics, version 23.0 (SPSS, Inc., Chicago, USA) was applied for statistical analyses.

## Results

### Demographic and baseline information

**Fig. 1:**
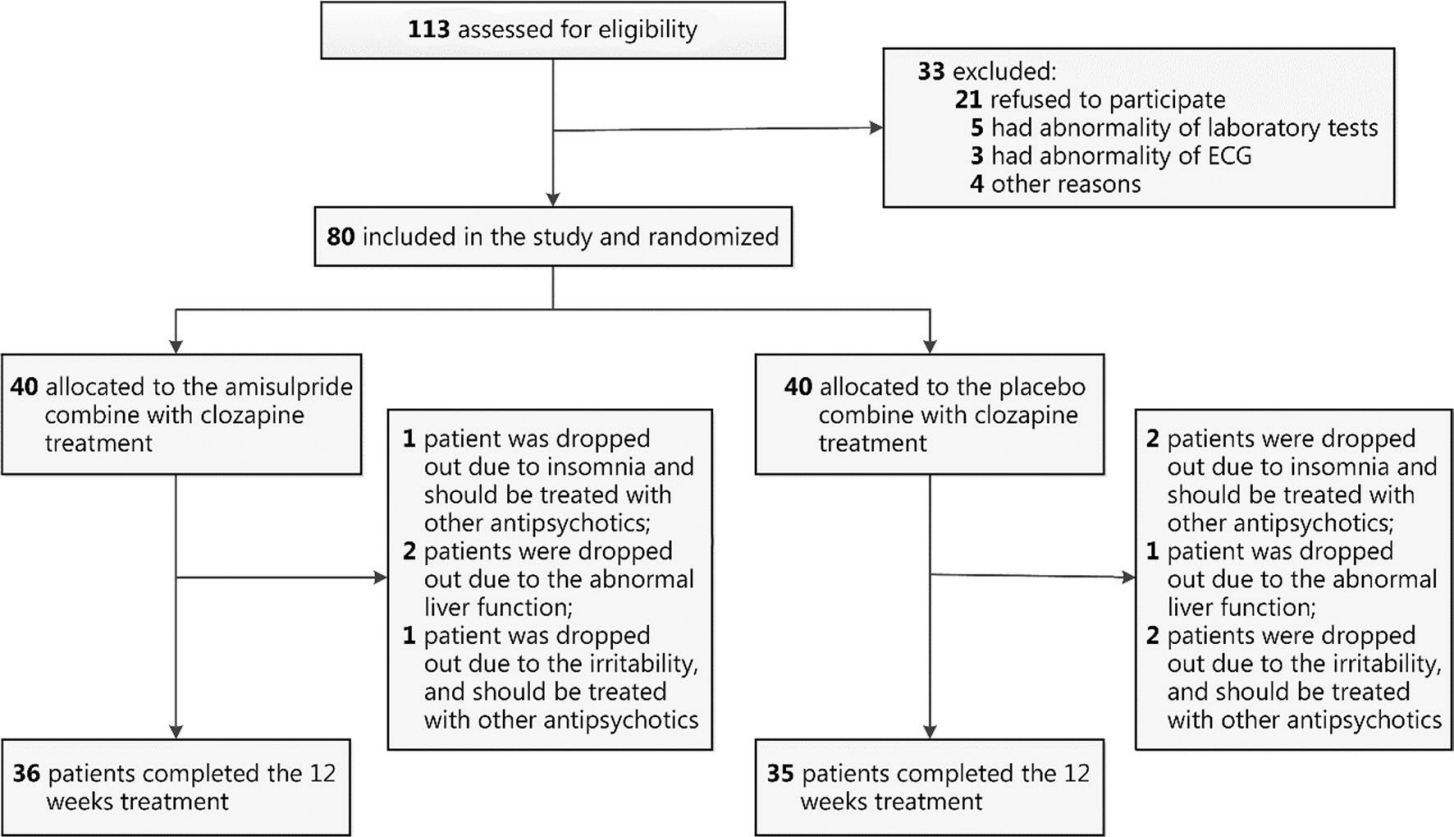
Treatment study flowchart. A total of 113 participants were assessed for eligibility, 80 were recruited and randomly assigned to one of the groups. Among these patients, 36 in the amisulpride group, while 35 in the placebo group completed the 12-week trial

Among the 113 participants assessed for eligibility, 80 were recruited and randomly assigned to one of the groups (Fig. 1). Among these patients, 78 completed the 6-week trial, and 71 completed the 12-week trial. At week 6, one patient (2.5%) in the amisulpride group and one in the placebo group dropped out. At week 12, 3 patients (7.5%) in the amisulpride group and 4 (10.0%) in the placebo group dropped out. The average dose of amisulpride in the amisulpride group was 771.4 mg/d at the end of 12-week. As shown in Table 1, at baseline, except for BMI (*F* = 4.85, *P* = 0.03), there was no significant difference in any demographic or clinical characteristics (PANSS, RBANS, SANS, and CGI scores) between the two groups (*P* > 0.05). The amisulpride group had a higher BMI than the placebo group. Therefore, BMI was adjusted in the subsequent statistical analysis. There was no difference in the clozapine dose or serum clozapine levels at baseline between the amisulpride and the placebo groups (*P* > 0.05). Furthermore, after adjusting BMI and baseline clozapine dose, RM ANOVA showed no group × time effect, time effect, or group effect on serum clozapine levels (*P* > 0.05), indicating that there was no difference in the change of serum clozapine levels after 12 weeks of treatment. In addition, there was no difference in demographic or clinical characteristics between dropouts and completers (*P* > 0.05).

**Table 1:** Demographic and clinical data of amisulpride and placebo groups at baseline

Item	Amisulpride (*n* = 40)	Placebo (*n* = 40)	*X*^2^ or *F*(*P*)	Dropouts (*n* = 9)	Completers (*n* = 71)	*X*^2^ or *F*(*P*)
Age (years, mean ± SD)	46.60 ± 9.36	47.95 ± 7.20	0.52 (0.48)	45.33 ± 4.80	47.52 ± 8.67	0.54 (0.46)
Sex (*n*, male/female)	19/21	22/18	0.45 (0.50)	4/5	36/35	0.08 (0.78)
BMI (kg/m^2^, mean ± SD)	24.76 ± 3.47	23.06 ± 2.84	4.85 (0.03)	23.00 ± 0.99	24.02 ± 3.43	1.05 (0.31)
Education (years, mean ± SD)	11.48 ± 2.46	11.10 ± 2.26	0.27 (0.61)	10.78 ± 1.48	11.35 ± 2.44	0.75 (0.39)
Age of onset (years, mean ± SD)	22.70 ± 5.32	23.45 ± 6.26	0.26 (0.60)	23.89 ± 4.37	22.97 ± 5.95	0.38 (0.54)
Illness duration (years, mean ± SD)	23.05 ± 5.65	23.73 ± 6.45	0.19 (0.66)	24.38 ± 4.52	23.26 ± 6.22	0.39 (0.54)
Clozapine dose (mg/d, mean ± SD)	438.75 ± 32.99	447.50 ± 37.47	1.21 (0.28)	441.67 ± 30.62	443.31 ± 36.10	0.07 (0.80)
Serum clozapine level (ng/ml, mean ± SD)	478.05 ± 81.17	491.05 ± 80.17	0.56 (0.46)	484.11 ± 61.17	484.61 ± 82.90	0.01 (0.91)
PANSS total score (mean ± SD)	82.28 ± 8.54	79.53 ± 6.71	2.59 (0.11)	79.44 ± 4.33	81.08 ± 8.09	0.41 (0.53)
P subscore	19.40 ± 6.54	20.73 ± 3.90	1.44 (0.23)	19.11 ± 4.11	20.18 ± 5.54	0.30 (0.59)
N subscore	21.15 ± 7.45	18.98 ± 4.69	2.86 (0.10)	21.78 ± 4.21	19.85 ± 6.49	0.94 (0.34)
G subscore	41.50 ± 6.33	39.83 ± 3.55	2.17 (0.15)	38.67 ± 3.71	40.92 ± 5.29	1.91 (0.17)
RBANS total score (mean ± SD)	59.38 ± 7.71	60.28 ± 10.14	0.23 (0.64)	59.44 ± 5.73	59.87 ± 9.32	0.02 (0.89)
Immediate memory	59.43 ± 9.65	59.83 ± 10.68	0.05 (0.83)	60.33 ± 3.35	59.54 ± 11.25	0.04 (0.83)
Visuospatial/construction	67.50 ± 12.23	68.65 ± 11.97	0.11 (0.74)	68.11 ± 5.18	68.07 ± 12.67	0.001 (0.98)
Language	71.15 ± 14.02	71.63 ± 13.86	0.08 (0.78)	70.22 ± 5.87	71.54 ± 14.58	0.07 (0.79)
Attention	73.65 ± 9.68	73.75 ± 11.52	0.26 (0.61)	72.89 ± 5.13	73.80 ± 11.41	0.04 (0.84)
Delayed memory	57.93 ± 7.71	61.03 ± 10.14	1.52 (0.22)	59.00 ± 6.93	59.54 ± 10.11	0.02 (0.89)
SANS (mean ± SD)	45.18 ± 15.38	43.13 ± 14.36	0.36 (0.55)	42.56 ± 6.80	44.38 ± 11.53	0.12 (0.73)
CGI-S (mean ± SD)	5.03 ± 0.70	4.90 ± 0.71	0.49 (0.49)	5.11 ± 0.78	4.94 ± 0.70	0.42 (0.52)
CGI-I (mean ± SD)	4.06 ± 0.22	4.03 ± 0.16	0.17 (0.68)	4.11 ± 0.33	4.03 ± 0.17	1.30 (0.26)
CGI-E (mean ± SD)	13.60 ± 0.50	13.58 ± 0.50	0.34 (0.56)	13.44 ± 0.53	13.61 ± 0.49	0.59 (0.45)

*BMI* body mass index, *PANSS* Positive and Negative Syndrome Scale, *P* positive symptom, *N* negative symptom, *G* general psychopathology, *RBANS* Repeatable Battery for the Assessment of Neuropsychological Status, *CGI* Clinical Global Impression scale, *CGI-S* CGI severity, *CGI-I* CGI improvement, *CGI-E* CGI efficacy

### Effect of amisulpride augmentation therapy on PANSS scores

**Fig. 2:**
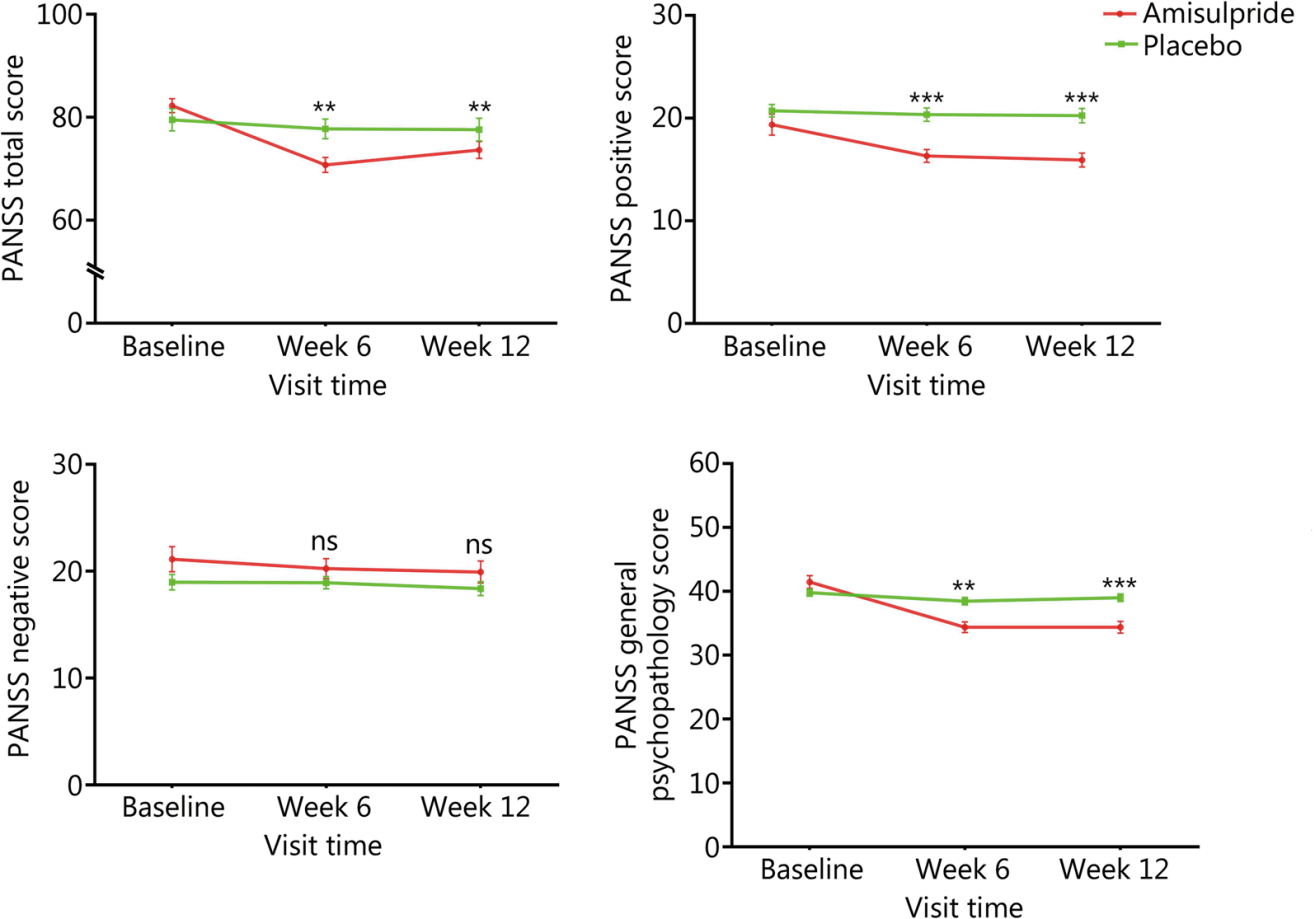
Effect of amisulpride augmentation therapy on Positive and Negative Syndrome Scale (PANSS) scores. At week 6 and 12, the amisulpride group displayed lower PANSS total score, positive symptom subscore, and general psychopathology subscore compared with the placebo group (week 6:*F* = 11.18, *P* = 0.001, *P*_Bonferroni_ = 0.004, Cohen’s* d* = 0.88; *F* = 16.63, *P* < 0.0001, *P*_Bonferroni_ < 0.0001, Cohen’s* d* = 0.99; *F* = 12.56, *P* = 0.001, *P*_Bonferroni_ = 0.004, Cohen’s* d* = 0.89. week 12:*F* = 11.34, *P* = 0.001, *P*_Bonferroni_ = 0.004, Cohen’s* d* = 0.45; *F* = 17.10, *P* < 0.0001, *P*_Bonferroni_ < 0.0001, Cohen’s* d* = 0.97; *F* = 14.00, *P* < 0.0001, *P*_Bonferroni_ < 0.001, Cohen’s* d* = 0.92). **P < 0.01, ***P < 0.001; ns non-significant

RM MANOVA was first conducted using PANSS subscales and total score as the outcome measurement and BMI as the covariate, and showed a significant group × time effect (Wilks’ lambda *F* = 10.50, *P* < 0.0001). Then, RM ANOVA showed group × time effects on PANSS total score (Wilks’ lambda *F* = 11.75, *P* < 0.001), positive symptom subscore (Wilks’ lambda *F* = 3.66, *P* = 0.03) and general psychopathology subscore (Wilks’ lambda *F* = 9.03, *P* < 0.001) (Table 2). Next, after adjusting for BMI, age, sex, disease course, baseline PANSS scores, and baseline clozapine serum levels, an ANCOVA was used to examine the group difference in PANSS total and subscale scores at week 6 and week 12, respectively. As shown in Fig. 2 at week 12, amisulpride group displayed lower PANSS total score, positive symptom subscore, and general psychopathology subscore compared with placebo group (*P*_Bonferroni_ = 0.004, Cohen’s *d* = 0.45; *P*_Bonferroni_ < 0.0001, Cohen’s *d* = 0.97; *P*_Bonferroni_ < 0.001, Cohen’s *d* = 0.92; respectively). At week 6, amisulpride group displayed lower PANSS total score, positive symptom subscore and general psychopathology subscore compared with placebo group (*P*_Bonferroni_ = 0.004, Cohen’s *d* = 0.88; *P*_Bonferroni_ < 0.0001, Cohen’s *d* = 0.99; *P*_Bonferroni_ = 0.004, Cohen’s *d* = 0.89; respectively).

**Table 2:** The scores of PANSS, RBANS, SANS and CGI at baseline, week 6 and week 12 follow-up in amisulpride and placebo groups

Item	Baseline		Week 6		Week 12		Group *F*(*P*)	Time *F*(*P*)	Group × time *F*(*P*)
	Amisulpride	Placebo	Amisulpride	Placebo	Amisulpride	Placebo			
PANSS total score	82.28 ± 8.54	79.53 ± 6.71	70.75 ± 9.47	77.75 ± 5.99	73.68 ± 10.35	77.63 ± 6.86	3.51 (0.07)	1.25 (0.29)	11.75 (< 0.001)
P subscore	19.40 ± 6.54	20.73 ± 3.90	16.33 ± 3.94	20.35 ± 4.15	15.93 ± 4.36	20.25 ± 4.56	10.99 (0.001)	0.09 (0.92)	3.66 (0.03)
N subscore	21.15 ± 7.45	18.98 ± 4.69	20.27 ± 5.95	18.93 ± 3.74	19.95 ± 6.63	18.38 ± 4.16	3.08 (0.08)	0.73 (0.49)	0.91 (0.41)
G subscore	41.48 ± 6.33	39.83 ± 3.55	34.40 ± 5.38	38.48 ± 3.59	34.38 ± 6.01	39.00 ± 3.76	5.43 (0.02)	1.27 (0.29)	9.03 (< 0.001)
RBANS total score	59.38 ± 7.71	60.28 ± 10.14	63.20 ± 8.88	61.75 ± 10.72	65.03 ± 8.31	61.25 ± 9.98	0.48 (0.49)	1.49 (0.23)	3.54 (0.03)
Immediate memory	59.43 ± 9.65	59.83 ± 11.68	61.83 ± 9.26	59.50 ± 10.66	63.50 ± 8.68	60.63 ± 9.83	0.52 (0.47)	0.07 (0.93)	2.11 (0.13)
Visuospatial/construction	67.50 ± 12.23	68.65 ± 11.97	73.30 ± 13.93	70.33 ± 14.29	72.43 ± 12.02	68.35 ± 12.22	0.29 (0.60)	1.06 (0.35)	1.63 (0.20)
Language	71.15 ± 14.02	71.63 ± 13.86	78.05 ± 11.66	74.55 ± 12.99	81.20 ± 10.20	72.38 ± 12.68	2.35 (0.13)	0.92 (0.40)	5.49 (0.006)
Attention	73.65 ± 11.58	73.75 ± 13.94	76.25 ± 11.68	72.55 ± 11.89	74.25 ± 10.90	73.33 ± 11.57	0.16 (0.69)	2.33 (0.10)	2.37 (0.10)
Delayed memory	57.93 ± 9.68	61.03 ± 11.52	62.15 ± 10.14	63.38 ± 13.06	61.48 ± 9.72	63.05 ± 13.37	0.60 (0.44)	0.47 (0.63)	0.86 (0.43)
SANS	45.18 ± 15.38	43.13 ± 14.36	44.78 ± 14.88	44.13 ± 13.56	43.88 ± 13.14	44.68 ± 14.78	0.20 (0.66)	1.03 (0.36)	2.83 (0.07)
CGI-S	5.03 ± 0.70	4.90 ± 0.71	4.20 ± 0.61	4.63 ± 0.59	4.20 ± 0.61	4.78 ± 0.66	3.50 (0.07)	1.26 (0.29)	10.85 (< 0.001)
CGI-I	4.05 ± 0.22	4.03 ± 0.16	3.48 ± 0.92	3.90 ± 0.55	3.48 ± 0.96	4.03 ± 0.16	4.41 (0.04)	0.17 (0.85)	4.16 (0.02)
CGI-E	13.60 ± 0.50	13.58 ± 0.50	10.88 ± 3.58	12.98 ± 1.92	10.88 ± 3.58	13.58 ± 0.50	15.38 (< 0.001)	0.52 (0.60)	12.17 (< 0.001)

Data were expressed as mean ± SD. *PANSS* Positive and Negative Syndrome Scale, *P* positive symptom, *N* negative symptom, *G* general psychopathology, *RBANS* Repeatable Battery for the Assessment of Neuropsychological Status, SANS Scale for the Assessment of Negative Symptoms, *CGI* Clinical Global Impression scale, *CGI-S* CGI severity, *CGI-I* CGI improvement, *CGI-E* CGI efficacy

### Effect of amisulpride augmentation therapy on treatment response rate

ITT analysis showed that, at week 12, the response rate of the amisulpride group (10 patients, 25%) was higher than that of the placebo group (2 patients, 5%) (*χ*^2^ = 6.28, *P* = 0.01, *OR* = 6.33, 95% CI 1.29–31.12). After adjusting for BMI, age, sex, disease course, and baseline clozapine serum levels, it also showed a significant between-group difference (B = 1.82, Wald’s statistics = 4.06, *P* = 0.04, *OR* = 6.15, 95% CI 1.06–36.03), the amisulpride group had a higher response rate than the placebo group.

### Effect of amisulpride augmentation therapy on cognitive function

RM MANOVA showed a significant group × time effect (Wilks’ lambda *F* = 4.64; *P* = 0.01) on RBANS scores. After adjusting for BMI, an RM ANOVA was applied for RBANS total and subscale scores, respectively. As shown in Table 2, there was a group × time effect of RBANS total score and language score (Wilks’ lambda *F* = 3.54, *P* = 0.03; Wilks’ lambda *F* = 5.49, *P* = 0.006).

**Fig. 3:**
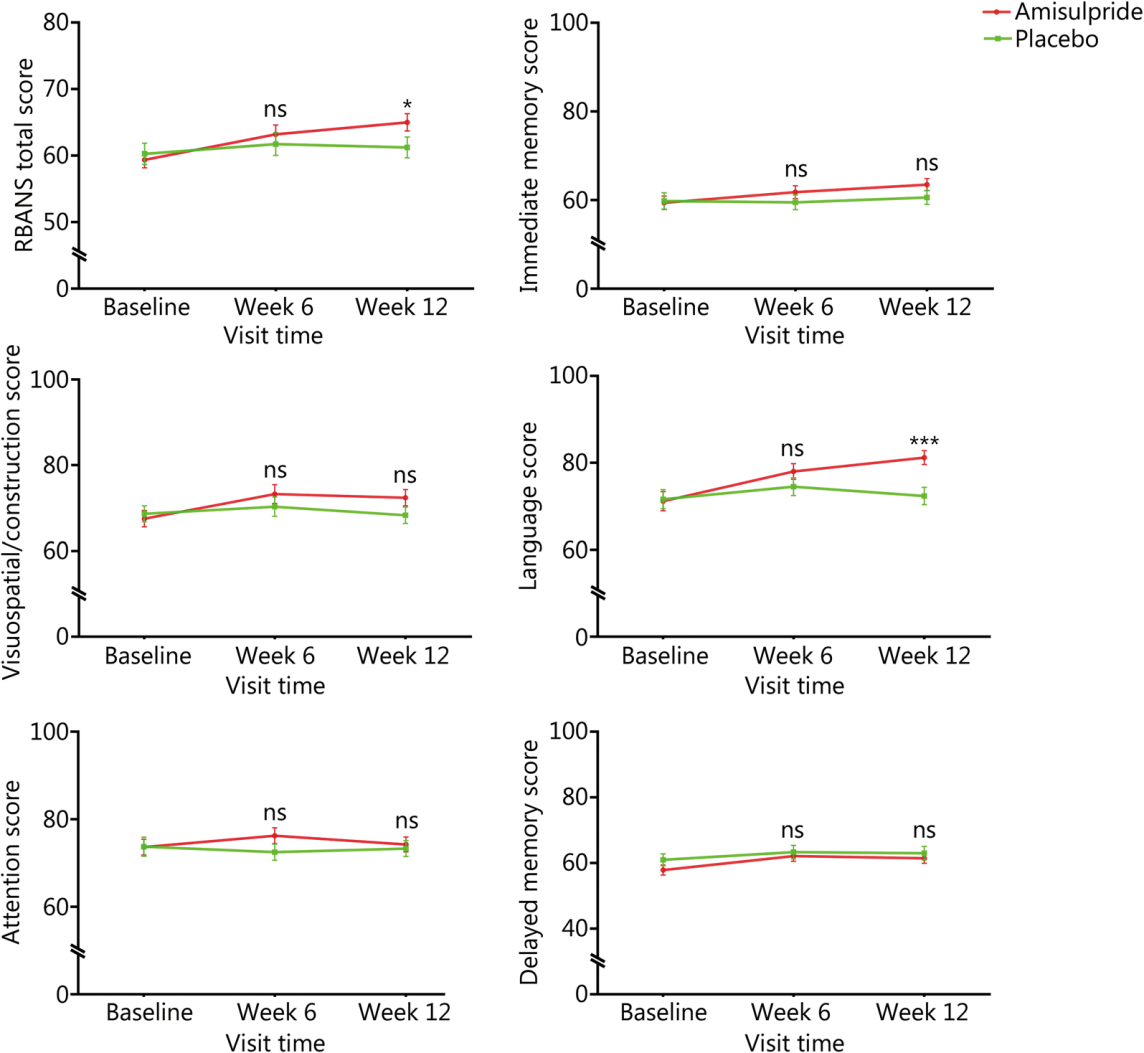
Effect of amisulpride augmentation therapy on Repeatable Battery for the Assessment of Neuropsychological Status (RBANS) scores. At week 12, the amisulpride group displayed higher RBANS total and language scores compared with placebo group (*F* = 6.14, *P* = 0.01, Cohen’s *d* = 0.41; *F* = 14.82, *P* < 0.0001, Cohen’s *d* = 0.77). However, only the difference in language score remained significant after Bonferroni correction (*P*_Bonferroni_ < 0.001). At week 6, there were no between-group differences in RBANS total score or language score (*F* = 2.52, *P* = 0.12; *F* = 3.14, *P* = 0.08). *P < 0.5, ***P < 0.001; ns non-significant

Then, after adjusting the baseline score and other clinical covariates, an ANCOVA was applied to examine the group differences in the RBANS total score and language score at week 6 and week 12, respectively. As shown in Fig. 3, at week 12, the amisulpride group displayed higher RBANS total and language scores compared with placebo group (*P* = 0.01, Cohen’s *d* = 0.41; *P* < 0.0001, Cohen’s *d* = 0.77). However, only the difference in language score remained significant after Bonferroni correction (*P*_Bonferroni_ < 0.001). At week 6, there were no between-group differences in RBANS total score or language score (*P* = 0.12; *P* = 0.08).

### Effect of amisulpride augmentation therapy on SANS and CGI scores

**Fig. 4:**
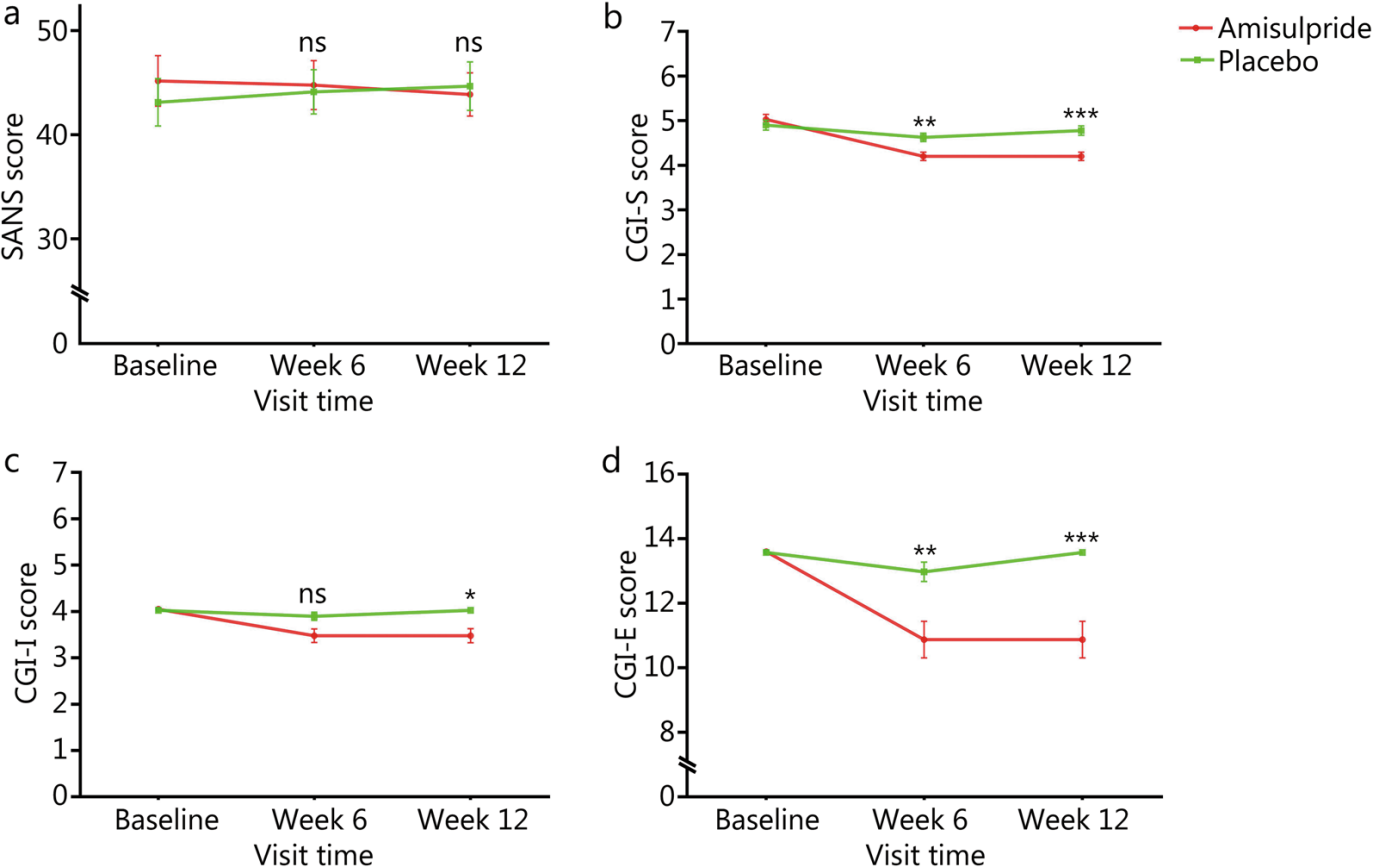
Effect of amisulpride augmentation therapy on the scores of Scale for the Assessment of Negative Symptoms (SANS) and Clinical Global Impression (CGI). **a** SANS score. **b–d** CGI-S, CGI-I, and CGI-E scores. SANS score showed no difference between two groups at week 12 or week 6 (**a**). At week 12, the amisulpride group had lower CGI-S, CGI-I and CGI-E scores than the placebo group (*F* = 20.37, *P* < 0.0001, Cohen’s *d* = 0.91; *F* = 5.75, *P* = 0.02, Cohen’s *d* = 0.80; *F* = 19.02, *P* < 0.0001, Cohen’s *d* = 1.06; respectively). However, after Bonferroni correction, only CGI-S and CGI-E scores still showed significant between-group differences (both *P*_Bonferroni_ < 0.0001) (**b–d**). At week 6, the amisulpride group had lower CGI-S and CGI-E scores than the placebo group (*F* = 10.93, *P* = 0.001, *P*_Bonferroni_ = 0.003, Cohen’s *d* = 0.72; *F* = 8.98, *P* = 0.004, *P*_Bonferroni_ = 0.01, Cohen’s *d* = 0.73) (**b–d**). *P < 0.05, **P < 0.01, ***P < 0.001; ns non-significant

An RM ANOVA was performed on SANS, CGI-S, CGI-I, and CGI-E scores, after controlling for BMI as a covariate. As shown in Table 2, there were group × time effects on CGI-S (Wilks’ lambda *F* = 10.85, *P* < 0.001), CGI-I (Wilks’ lambda *F* = 4.16, *P* = 0.02) and CGI-E (Wilks’ lambda *F* = 12.17, *P* < 0.001) scores. However, there was no significant group × time effect on the SANS score (Fig. 4a).

Next, an ANCOVA was conducted to examine the group differences in the CGI-S, CGI-I, and CGI-E scores at week 6 and week 12, respectively, after adjusting for baseline scores and other clinical covariates. As shown in Fig. 4b-d, at week 12, the amisulpride group had lower CGI-S, CGI-I and CGI-E scores than the placebo group (*P* < 0.0001, Cohen’s *d* = 0.91; *P* = 0.02, Cohen’s *d* = 0.80; *P* < 0.0001, Cohen’s *d* = 1.06; respectively). However, after Bonferroni correction, only CGI-S and CGI-E scores still showed significant between-group differences (both *P*_Bonferroni_ < 0.0001). At week 6, the amisulpride group had lower CGI-S and CGI-E scores than the placebo group (*P*_Bonferroni_ = 0.003, Cohen’s *d* = 0.72; *P*_Bonferroni_ = 0.01, Cohen’s *d* = 0.73).

### Treatment side effects and safety

As shown in Table 3, after adjusting for BMI as a covariate, RM ANOVA showed no significant group × time effect, main time effect, or group effect on the TESS total score (*P* > 0.05). In addition, after Bonferroni correction, there were no significant group × time effects on BMI, QTc intervals, or laboratory measurements (*P*_Bonferroni_ > 0.05).

**Table 3:** TESS score, BMI, QT interval and laboratory parameters at baseline, week 6 and week 12 in amisulpride and placebo groups

Item	Baseline		Week 6		Week 12		Group *F*(*P*)	Time *F*(*P*)	Group × time *F*(*P*)
	Amisulpride	Placebo	Amisulpride	Placebo	Amisulpride	Placebo			
TESS score^a^	4.83 ± 3.81	4.93 ± 3.50	6.40 ± 4.82	5.40 ± 3.59	6.30 ± 4.76	5.55 ± 3.63	2.98 (0.42)	0.04 (0.96)	0.79 (0.38)
BMI	24.76 ± 3.47	23.06 ± 2.84	24.82 ± 3.39	23.10 ± 2.81	24.96 ± 3.45	23.19 ± 2.95	5.24 (0.03)	1.50 (0.23)	0.55 (0.58)
WBC^a^ (× 10^9^)	6.00 ± 1.21	6.53 ± 1.91	6.11 ± 1.45	6.59 ± 1.52	6.06 ± 1.49	7.06 ± 1.71	2.96 (0.09)	0.08 (0.92)	1.14 (0.33)
Neutrophils^a^ (× 10^9^)	3.77 ± 0.81	4.15 ± 1.62	3.57 ± 1.22	4.12 ± 1.23	3.66 ± 1.00	4.56 ± 1.60	4.45 (0.04)	0.74 (0.48)	1.40 (0.25)
RBC^a^ (× 10^12^)	4.11 ± 0.53	4.26 ± 0.51	4.14 ± 0.55	4.24 ± 0.50	4.20 ± 0.53	4.18 ± 0.46	0.68 (0.41)	0.04 (0.96)	1.81 (0.17)
Hb^a^ (g/L)	121.43 ± 13.60	128.85 ± 13.51	123.00 ± 14.33	129.62 ± 11.06	123.54 ± 14.16	129.62 ± 12.54	4.67 (0.03)	2.67 (0.08)	0.35 (0.70)
PLT^a^ (× 10^9^)	240.63 ± 57.46	261.26 ± 77.67	239.54 ± 63.58	261.59 ± 68.07	248.97 ± 69.62	252.59 ± 73.09	1.31 (0.26)	1.28 (0.28)	1.84 (0.17)
ALT^a^ (U/L)	16.80 ± 10.57	19.97 ± 12.87	18.09 ± 13.33	19.97 ± 12.98	18.46 ± 11.64	21.88 ± 11.60	1.84 (0.18)	0.19 (0.83)	0.25 (0.78)
AST^a^ (U/L)	20.46 ± 6.35	23.38 ± 10.05	21.37 ± 9.81	19.85 ± 5.68	18.29 ± 4.96	21.85 ± 7.37	1.99 (0.16)	0.14 (0.87)	4.52 (0.02)
BUN^a^ (mmol/L)	4.49 ± 1.20	4.03 ± 1.27	4.31 ± 0.90	4.35 ± 1.20	4.34 ± 1.39	4.18 ± 1.09	1.25 (0.27)	0.18 (0.84)	1.93 (0.15)
Cr^a^ (μmol/L)	62.74 ± 11.52	73.68 ± 15.22	63.34 ± 11.14	69.91 ± 19.03	62.17 ± 11.27	70.32 ± 16.36	6.31 (0.01)	0.29 (0.75)	2.01 (0.14)
Ur^a^ (μmol/L)	292.86 ± 91.53	352.26 ± 122.22	283.91 ± 90.78	359.24 ± 105.86	281.57 ± 98.38	333.00 ± 90.51	6.90 (0.01)	0.74 (0.48)	2.05 (0.14)
Glucose^a^ (mmol/L)	5.11 ± 0.93	5.59 ± 2.02	5.29 ± 0.89	5.26 ± 0.83	5.34 ± 1.00	5.47 ± 1.02	0.45 (0.51)	1.09 (0.34)	0.93 (0.40)
TG^a^ (mmol/L)	1.31 ± 0.53	1.59 ± 0.66	1.54 ± 0.74	1.74 ± 0.67	1.31 ± 0.68	1.62 ± 0.95	3.21 (0.08)	0.50 (0.61)	0.42 (0.66)
TC^a^ (mmol/L)	4.03 ± 0.71	4.18 ± 0.94	4.03 ± 0.79	4.24 ± 0.86	4.03 ± 0.86	4.06 ± 0.65	0.11 (0.74)	3.14 (0.05)	1.93 (0.15)
HDLC^a^ (mmol/L)	1.23 ± 0.43	1.18 ± 0.39	1.34 ± 0.48	1.18 ± 0.39	1.34 ± 0.48	1.12 ± 0.33	3.19 (0.08)	0.87 (0.43)	2.00 (0.14)
LDLC^a^ (mmol/L)	2.11 ± 0.76	2.32 ± 0.95	2.23 ± 0.69	2.38 ± 0.74	2.17 ± 0.71	2.38 ± 0.70	0.81 (0.37)	0.31 (0.74)	0.10 (0.90)
QTc interval^a^ (ms)	384.80 ± 30.67	411.79 ± 31.73	381.03 ± 29.58	413.24 ± 32.76	389.14 ± 31.77	408.41 ± 40.47	10.75 (0.002)	1.18 (0.31)	3.04 (0.06)

Data were expressed as mean ± SD. ^a^Adjusting for BMI [repeated-measures analysis of variance (RM ANOVA) with BMI as a covariate]. *TESS* Treatment Emergent Symptom Scale, *BMI* body mass index, *WBC* white blood cell, *RBC* red blood cell, *Hb* hemoglobin, *PLT* platelet, *ALT* alaninetransaminase, *AST* aspartate transaminase, *BUN* blood urea nitrogen, *Cr* creatinine, *Ur* uric acid, *TG* triglyceride, *TC* total cholesterol, *HDLC* high density lipoprotein, *LDLC* low density lipoprotein, *QTc* corrected QT

At week 12, the most common adverse effects were mild in both groups, including dry mouth, constipation, EPS, gastrointestinal reactions, saliva, hypersomnia, insomnia, and headache. There was no significant difference in the incidence of side effects between the amisulpride group and the placebo group (*P* > 0.05).

## Discussion

This randomized, double-blind, placebo-controlled trial study suggests that amisulpride augmentation therapy can safely improve the clinical symptoms and cognitive function of CTRS patients. Compared with the placebo group, the positive and general psychopathological symptoms of CTRS patients in the amisulpride augmentation group continued to improve at week 6 and week 12. Moreover, the 12-week amisulpride augmentation therapy increased the response rate compared to placebo. Also, compared with the placebo, the CGI-S and CGI-E scores of CTRS patients with amisulpride augmentation therapy were significantly reduced. Our results are partially consistent with a relatively small sample size (*n* = 16) open-label non-randomized study, which found that amisulpride augmentation therapy improved the positive symptoms of schizophrenia partially responded to clozapine [32].

The theory that amisulpride enhances the efficacy of clozapine is based on the fact that the receptor profiles of these two drugs are complementary. Among those who do not respond to clozapine, clozapine monotherapy may not reach the level of D2 receptor blockade [33, 34], because the level of D2 receptor blockade needs to be about 80% to produce a significant response [13, 35]. In patients who do not respond to clozapine monotherapy, the selective effects of amisulpride in the mesolimbic system may cause D2 receptors to be blocked at the therapeutic level. In addition, amisulpride appears to affect 5HT-7 receptors [36] and presynaptic autoreceptors, which may affect the regulation of endogenous dopamine production [37]. Furthermore, D3 receptors are located in the nucleus accumbens and cerebral cortex, and are associated with neural circuits implicated in schizophrenia [38, 39]. A meta-analysis involving more than 2500 patients showed a slight but significant correlation between D3 receptor coding sequence polymorphisms and susceptibility to schizophrenia [40]. Previous studies have shown that selective D3 antagonists may be effective antipsychotic agents for the treatment of schizophrenia. Because of their anatomical distribution in the ventral striatum [41], their locomotor adverse effects, including extrapyramidal side effects and catalepsy, may be negligible [42–44]. For example, a 6-week randomized, double-blind, placebo-controlled trial confirmed the efficacy and safety of D3 antagonists for improving acute exacerbations of schizophrenia [45]. It has also been reported that high D2 antagonism or higher doses of antipsychotic drugs, which are more likely to over-occupy D2 receptors, may increase the risk of secondary negative symptoms [46]. However, our study did not find any effects of amisulpride augmentation therapy on the negative symptoms of CTRS patients based on the PANSS negative subscale and the SANS assessment, which was consistent with the study of Barnes and their colleagues [19]. In clinical practice, negative symptoms of schizophrenia are usually stable and difficult to treat. Amisulpride has been proved to treat schizophrenia patients with predominantly negative symptoms and the approved dose is 50–300 mg/d [47]. In our study, the dose of amisulpride has exceeded 400 mg/d since the beginning of week 3, and we did not perform PANSS scale assessments at the endpoint of week 2. In addition, the high dopamine blockade effect, caused by clozapine and high doses of amisulpride in our study, may induce secondary negative symptoms causing insignificant reductions in negative scores [47].

Previous evidence suggests that amisulpride can improve the cognitive function of schizophrenia patients [48, 49]. However, few studies have examined the effects of amisulpride combined with clozapine on the cognitive function of schizophrenia patients. Park et al. [50] previously reported that amisulpride augmentation therapy improved the working memory of schizophrenia patients treated with aripiprazole. Recently, Molina et al. [51] revealed that the combined use of amisulpride and quetiapine improved both clinical symptoms and cognitive function, especially the executive function of TRS. In this study, we found that amisulpride augmentation therapy also improved the cognitive performance of CTRS patients, particularly language function.

Picture naming and semantic fluency tasks are the RBANS items used in this study to test the language domain. Similar to our research, Salmazo-Silva et al. [52] also used image naming and semantic fluency tasks to assess language abilities, but they employed Parkinson’s disease as their target ailment. Language and perception disorders are the core cognitive impairment symptoms in schizophrenia [53]. The underlying mechanism may be related to the antagonistic effects of amisulpride on D2, D3, and 5-HT7 receptors. For example, previous evidence showed that resting blood flow in the hippocampus of patients with schizophrenia was abnormally increased [54], indicating an increase in resting metabolism in this region [55, 56]. Tregellas et al. [57] found that the resting hyperactivity of the hippocampus strongly relates to cognitive deficits in schizophrenia patients. Interestingly, dopamine D2 antagonists have been shown to reverse the abnormal increase in hippocampal blood flow in patients with schizophrenia [58]. In addition, Shin et al. [59] found that D2 receptor antagonism may improve the working memory function of schizophrenia patients. Previous evidence suggests that 5-HT7 receptor antagonists may affect neuronal morphology [60, 61] and stimulate hippocampal neurogenesis [62, 63], related to schizophrenia and cognitive function. In addition, preclinical studies using a rat model of schizophrenia-like cognitive impairment have demonstrated that 5-HT7 receptor antagonists can improve pro-cognitive function, and that amisulpride can improve stress-related frontal lobe cognitive impairment [64]. Preclinical evidence shows that D3 antagonists can reverse the deficiency of dopamine tension in the prefrontal cortex [65], which may improve cognition [66–68].

In this study, there were no differences in side effects or safety between patients receiving amisulpride augmentation therapy and the placebo, which was partially consistent with a previous open-label, non-randomized study [32]. This outcome shows that amisulpride augmentation therapy improves positive symptoms of CTRS patients without exacerbating side effects. It is well established that long-term disease courses and antipsychotics, especially atypical antipsychotics, increase the prevalence of metabolic disorders [69]. In this study, a comparison between patients treated with clozapine alone and patients treated with clozapine plus amisulpride for 12 weeks showed that both groups had similar metabolic outcomes, including BMI, blood lipids, and fasting blood glucose. As for cardiac side effects, an overdose of amisulpride increases the risk of prolonged QTc, but the risk is low at therapeutic doses [70]. Our results indicate that the therapeutic doses of amisulpride augmentation therapy did not increase the risk of QTc interval prolongation in CTRS patients.

The study reported here had many strengths, including acceptable sample size, an appropriate observation period, and multidimensional efficacy and safety assessments. In addition, all plasma samples of participants were obtained, therefore, future study will be conducted to investigate the peripheral protein biomarkers for CTRS and treatment efficacy.

Some limitations of our study should be noted. First, the sample size is relatively small, and our findings should be verified in a larger sample that is drawn from multiple centers. Second, the patients included in this study had chronic conditions, so the results of this study cannot be generalized to other settings. Third, The follow-up time for cognitive function improvement is relatively short.

## Conclusions

In summary, our findings demonstrate that amisulpride augmentation therapy can safely improve clinical symptoms and cognitive function in CTRS patients. Amisulpride augmentation therapy has important clinical significance for the treatment of CTRS. Although the results of this study are promising, further multiple-center studies with larger sample sizes should be conducted to confirm the efficacy and safety of this treatment in different clinical settings.

## Data Availability

The datasets used and/or analysed during the current study are available from the corresponding author on reasonable request.

## Abbreviations

ANCOVA: Analysis of covariance
ANOVA: Analysis of variance
BMI: Body mass index
CGI: Clinical Global Impression scale
CGI-S: CGI severity
CGI-I: CGI improvement
CGI-E: CGI efficacy
CTRS: Clozapine-resistant treatment-refractory schizophrenia
DSM-IV: Diagnostic and Statistical Manual of Mental Disorders, Fourth Edition
ECG: Electrocardiogram
EPS: Extrapyramidal symptoms
ITT: Intent-to-treat
LOCF: Last-observation-carrying-forward
PANSS: Positive and Negative Syndrome Scale
QTc: Corrected QT
RBANS: Repeatable Battery for the Assessment of Neuropsychological Status
RM ANOVA: Repeated-measures analysis of variance
RM MANOVA: Repeated-measure multivariate analysis of variance
SANS: Scale for the Assessment of Negative Symptoms
TESS: Treatment Emergent Symptom Scale
TRS: Treatment-resistant schizophrenia
